# Fischer-344 *Tp53*-knockout rats exhibit a high rate of bone and brain neoplasia with frequent metastasis

**DOI:** 10.1242/dmm.025767

**Published:** 2016-10-01

**Authors:** Sarah A. Hansen, Marcia L. Hart, Susheel Busi, Taybor Parker, Angela Goerndt, Kevin Jones, James M. Amos-Landgraf, Elizabeth C. Bryda

**Affiliations:** 1 Department of Veterinary Pathobiology, University of Missouri, Columbia, MI 65211, USA; 2 Rat Resource and Research Center, University of Missouri, Columbia, MI 65211, USA; 3 Departments of Orthopaedics and Oncological Sciences, Huntsman Cancer Institute, University of Utah, Salt Lake City, UT 84112, USA

**Keywords:** p53, Rat, Osteosarcoma, Sarcoma, Cancer

## Abstract

Somatic mutations in the *Tp53* tumor suppressor gene are the most commonly seen genetic alterations in cancer, and germline mutations in *Tp53* predispose individuals to a variety of early-onset cancers. Development of appropriate translational animal models that carry mutations in *Tp53* and recapitulate human disease are important for drug discovery, biomarker development and disease modeling. Current *Tp53* mouse and rat models have significant phenotypic and genetic limitations, and often do not recapitulate certain aspects of human disease. We used a marker-assisted speed congenic approach to transfer a well-characterized *Tp53-*mutant allele from an outbred rat to the genetically inbred Fischer-344 (F344) rat to create the F344-*Tp53^tm1(EGFP-Pac)Qly^/*Rrrc (F344-*Tp53*) strain. On the F344 genetic background, the tumor spectrum shifted, with the primary tumor types being osteosarcomas and meningeal sarcomas, compared to the hepatic hemangiosarcoma and lymphoma identified in the original outbred stock model. The Fischer model is more consistent with the early onset of bone and central nervous system sarcomas found in humans with germline *Tp53* mutations. The frequency of osteosarcomas in F344-*Tp53* homozygous and heterozygous animals was 57% and 36%, respectively. Tumors were highly representative of human disease radiographically and histologically, with tumors found primarily on long bones with frequent pulmonary metastases. Importantly, the rapid onset of osteosarcomas in this promising new model fills a current void in animal models that recapitulate human pediatric osteosarcomas and could facilitate studies to identify therapeutic targets.

## Abstract (precis)

**Editors' choice:** Transferring a *Tp53*-knockout allele from an outbred rat stock to the F344 inbred rat genetic background alters the spectrum of tumors, providing a model of early-onset brain and bone sarcomas.

## INTRODUCTION

Cancer researchers have extensively studied the *Tp53* gene, as it is the most frequently mutated gene in human cancers (Hollstein et al., 1991; Petitjean et al., 2007). Discovered in 1979 and labeled the ‘guardian of the genome’, the *Tp53* gene encodes a transcription factor (Tp53) that has tumor suppressor roles (Lane, 1992; Levine et al., 1991; Linzer et al., 1979). In response to DNA damage, the Tp53 protein specifically binds to DNA, transcribing genes that halt cell replication, initiate DNA repair and, at times, result in cell senescence or apoptosis (Mirza et al., 2003; Rivlin et al., 2011; Tokino and Nakamura, 2000). Mutations resulting in the absence of normal Tp53 in a cell lead to further genetic instability and improper cell cycle regulation. This ultimately results in the propagation of additional mutations, leading to malignancy (Lane, 1992). Although rare, germline mutations in *TP53* result in a condition known as Li-Fraumeni syndrome (LFS), a familial disorder characterized by the development of a wide variety of tumors in the first through third decades of life (Malkin et al., 1990). The most frequently identified tumors in LFS individuals are breast carcinomas, soft-tissue sarcomas, osteosarcomas, and central nervous system (CNS) tumors, although the spectrum varies with age as well as the specific mutation (Bougeard et al., 2015).

Recently, knowledge surrounding the biochemical and physiological processes of Tp53 has advanced more rapidly with the development of transgenic rodent models. Mice have been used to study both the loss of Tp53 function and dominant-negative forms of the protein (Donehower and Lozano, 2009; Rivlin et al., 2011). A potential drawback of many of the mouse models is the relatively high rate of malignant lymphoma in the Tp53-null state that is strongly dependent upon the genetic background (Donehower et al., 1995; Harvey et al., 1993). This leads to early morbidity and mortality and subsequently, due to the shortened lifespan, the full tumor spectrum seen in human LFS is not seen in the mouse. As a possible alternative rodent model, the rat has many advantages. The rat has a larger body size and more human-like metabolism than the mouse, making rats a better model species for preclinical drug and device development (Aitman et al., 2008). With recent advances in genome modification technologies, previous barriers to making genetically engineered rats no longer exist. In fact, in 2010, *Tp53* gene targeting was accomplished in the rat using some of these new technological advances (Tong et al., 2010). Currently, there are three well-characterized mutant rat models of *Tp53*, each developed in outbred stocks, and two additional inbred models that have yet to be extensively characterized (Table 1). These rat models show a decreased incidence of lymphoma and better representation of human cancers than many of the *Tp53* mouse models.

**Table 1.**
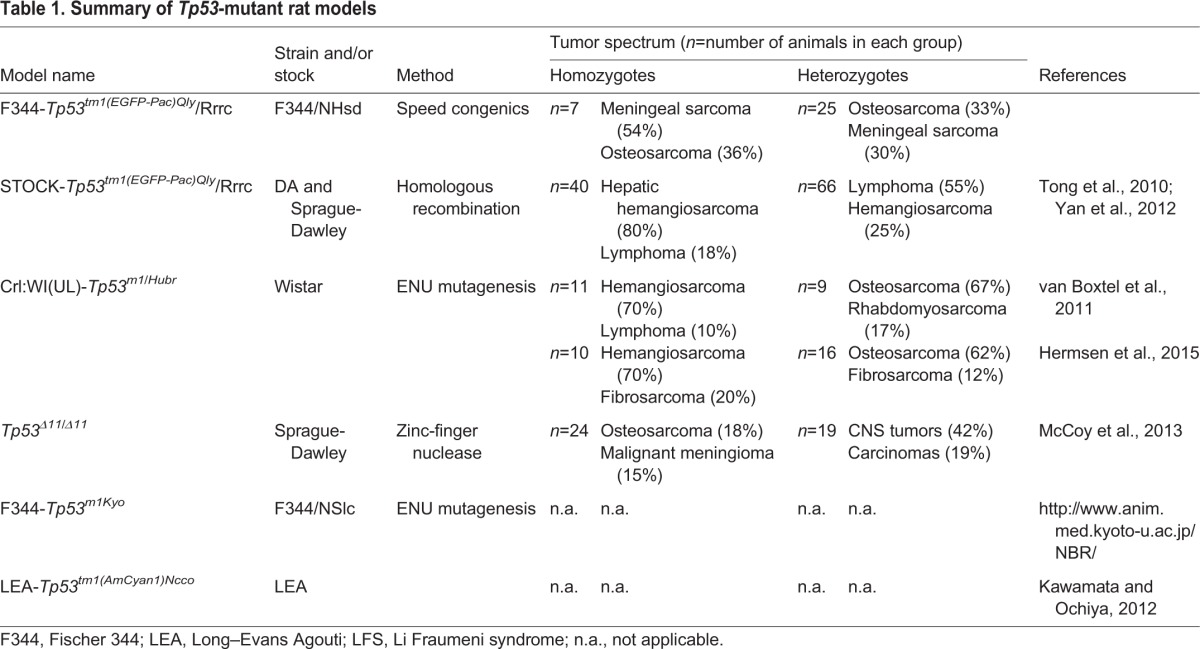
**Summary of *Tp53*-mutant rat models**

Often, Mendelian diseases are greatly influenced by the genetic background of the individual, creating highly variable phenotypes between individuals. *Tp53* mutations have been generated in a wide range of inbred mouse strains to investigate the influence of genetic background as a factor in phenotype variability (Donehower et al., 1995; Harvey et al., 1993). *Tp53*-deficient mice on a C57BL/6 background rarely develop mammary neoplasia, whereas BALB/c mice carrying the same mutant allele have a 55% incidence of mammary carcinoma in the heterozygous state (Kuperwasser et al., 2000). Rat models of human genetic disease similarly show phenotypic variability that depends on genetic background (Amos-Landgraf et al., 2007; Kantachuvesiri et al., 1999). Likewise, variability is seen across the spectrum of human disease categories, including but not limited to neurofibromatosis type 1, cystic fibrosis, breast cancer, Fanconi anemia, Li Fraumeni syndrome and hypertrophic cardiomyopathy (Boyle, 2007; Lopes et al., 2015; Metheny et al., 1995; Neveling et al., 2009; Nichols and Malkin, 2015; Szabo and King, 1995).

The outbred genetic nature of the existing rat *Tp53* knockouts might contribute to tumor variability, complicating reproducibility of studies. We transferred the *Tp53*-gene-knockout allele from an outbred stock to the Fischer-344 (F344) inbred strain of rat to stabilize the genetic background and determine if the genetic background would have an impact on the disease phenotype. We show that the phenotype of the inbred F344-*Tp53^tm1(EGFP-Pac)Qly^/*Rrrc (F344-*Tp53*) rat differs from that of previously reported *Tp53* mutants. On the inbred genetic background, there is a high incidence of brain tumors, and bone tumors that are accompanied by frequent pulmonary metastasis. This new strain provides a refined model for cancer research to investigate the role of *Tp53* in cancer initiation, propagation and metastasis.

## RESULTS

### Generation of a congenic Tp53 rat model on the Fischer-344 genetic background

**Fig. 1.**
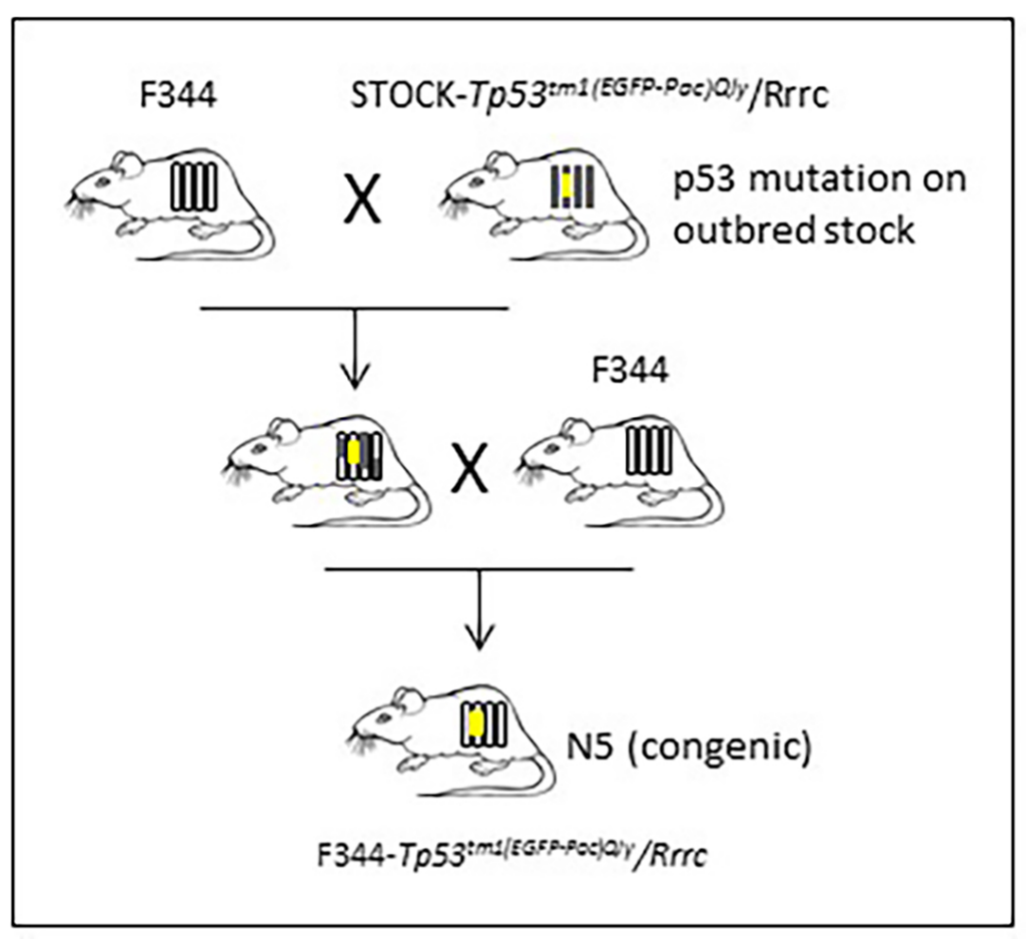
**Speed congenics.** An F344 congenic strain was generated using a marker-assisted microsatellite approach. Males heterozygous for the *Tp53* mutant allele that had the most F344 alleles at tested loci were backcrossed to F344 females. The strain was congenic at the N5 generation.

Beginning with the original STOCK-*Tp53^tm1(EGFP-Pac)Qly^/*Rrrc rat, we backcrossed to F344/NHsd using a marker-assisted speed congenic approach to transfer the *Tp53* mutation onto the F344 background (Fig. 1). *Tp53* heterozygous males carrying the highest number of tested loci that were homozygous for F344 alleles were selected at each generation to be backcrossed to F344 females. The process was repeated through five generations, resulting in a congenic strain that carries the *Tp53^tm1(EGFP-Pac^*^)^ null allele on a F344 genetic background. Once congenic, the strain was maintained by continued backcrossing to F344. Included in the final phenotype characterization were 25 heterozygous rats (12 male, 13 female) and seven homozygous rats (six male, one female), the progeny from matings of N5-N7 heterozygous *F344-Tp53^tm1(EGFP-Pac)Qly^/Rrrc* parents. Sixty-nine offspring were produced from heterozygous intercross matings (18 wild-type, 40 heterozygotes and 11 homozygous mutant animals). The genotypic ratios were as expected for Mendelian inheritance of an autosomal allele. However, there was a significant sex bias in the number of homozygous mutant animals such that ten males and only one female were recovered (Chi squared, *P*<0.03).

### F344*-Tp53* rats experience early-onset tumor development

**Fig. 2.**
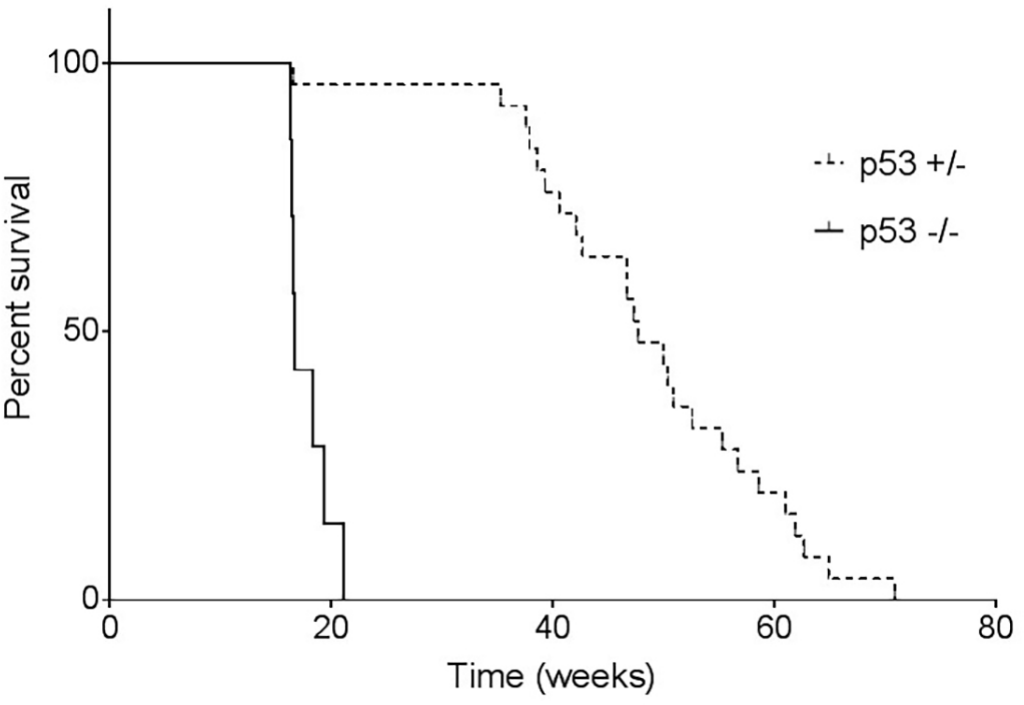
**Time to morbidity.** F344-*Tp53* rats were monitored daily and euthanized when signs of illness were identified: tumor presence, lethargy, dyspnea, abnormal mentation or a combination of the clinical signs. Homozygous rats had a median time to morbidity of 124 days (18 weeks), whereas heterozygous rats exhibited a median time to morbidity of 333 days (47 weeks) with a range of 115 to 438 days.

Previously reported *Tp53*-mutant mice and rats have a decreased lifespan compared to their wild-type counterparts owing to early tumor development (Donehower et al., 1995; McCoy et al., 2013; van Boxtel et al., 2011; Yan et al., 2012). We aged our animals until signs of tumor or illness were noted during daily evaluation, at which time we performed CO_2_ euthanasia and complete necropsy. The median time to morbidity for homozygous rats was 124 days (18 weeks), with a range of 16 to 21 weeks (Fig. 2). All homozygous animals developed one or more tumors within this time frame, including the single homozygous female that was euthanized at 16 weeks owing to dystocia (Table S1). The heterozygous rats developed disease, on average, by 47 weeks, with a range of 16 weeks to 62 weeks. One outlier was a heterozygous female rat euthanized at 16 weeks after presenting with exophthalmos, head tilt, ataxia and lethargy. She was determined to have a pituitary adenoma. We repeated genotyping on tissue from this animal and confirmed that it was indeed heterozygous, despite the unexpected early onset of morbidity. Overall, the F344 time to morbidity was longer than that reported for the original STOCK-*Tp53* line, where half of the STOCK-*Tp53* homozygotes developed tumors by 10 weeks, and half of heterozygotes were euthanized or died by 32 weeks (Yan et al., 2012).

### The tumor spectrum varies on different genetic backgrounds

**Fig. 3.**
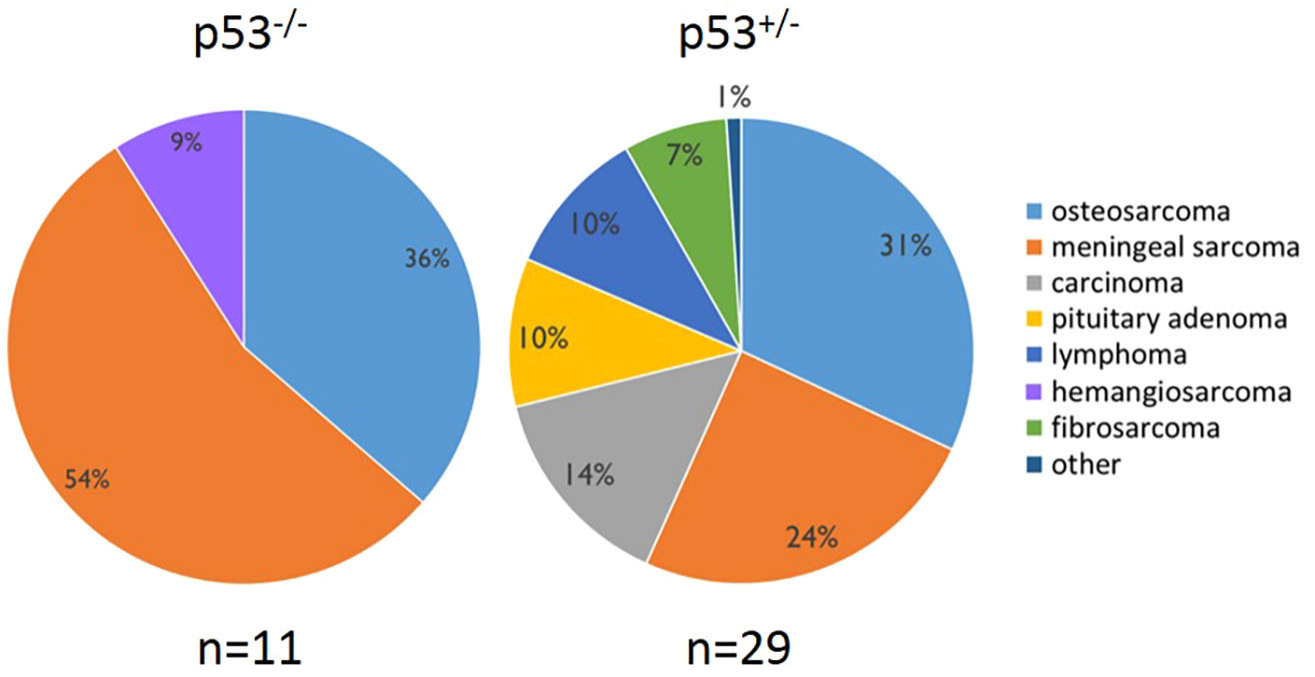
**F344-*Tp53* tumor phenotypes.** Rats homozygous or heterozygous for the *Tp53* mutation with outward signs of illness were euthanized and subjected to a full necropsy. Total numbers of tumors (*n*) identified in the 32 F344-*Tp53* rats are shown. Some animals had multiple tumor types. Predominant tumor types in both *Tp53*^−/−^ and *Tp53*^+/−^ rats were osteosarcoma and meningeal sarcoma, with heterozygous animals exhibiting a wider spectrum of tumors.

In our study, homozygous F344 rats developed primarily osteosarcomas, meningeal sarcomas or a combination of the two tumor types (Fig. 3). One hemangiosarcoma was identified in a superficial tumor on a homozygous animal also bearing an osteosarcoma and meningeal sarcoma (Table S1). Heterozygous rats experienced a wider range of tumor types, with 9 out of 25 (36%) animals developing osteosarcoma. Seven heterozygous rats (28%) had meningeal sarcomas. Three heterozygotes that had similar clinical presentations of labored breathing and a hunched posture were diagnosed with lymphoblastic lymphoma, exhibiting a space-occupying thymic mass as well as metastasis throughout the lungs, kidneys, spleen, liver, vasculature and lymph nodes throughout the body. Three heterozygous animals developed pituitary adenomas, of which two exhibited changes in mentation (head tilt, ataxia, moribund). The third was an incidental finding in an animal with an osteosarcoma of the tibia. Pituitary gland tumors have been noted to occur in aged (1-2 years old) wild-type F344 rats with a rate of 30.4% in males and 54.2% in females (Haseman et al., 1998). The STOCK-*Tp53* study reported only one brain tumor in mutant animals out of a large cohort, although the exact location within the brain was not specified (Yan et al., 2012). Three cases of lymphoma were seen in our study (9%), compared to 40% reported in the STOCK-*Tp53* mutants (Yan et al., 2012). Although the original STOCK-*Tp53* and F344-*Tp53* rats both developed primarily sarcomas, the target tissue was vastly different. For example, 80% of homozygous STOCK-*Tp53* knockout rats developed hepatic hemangiosarcoma, a tumor not seen in F344-*Tp53* rats in our study.

Two heterozygous F344-*Tp53* females presented with mammary adenocarcinoma, representing a similarity between the two models, in that 6 out of 31 female STOCK-*Tp53* heterozygous rats displayed mammary carcinomas, compared to 2 out of 13 cases of mammary adenocarcinoma in the F344-*Tp53* rats (Yan et al., 2012). Also identified in heterozygotes were cases of papillary cyst adenocarcinoma, fibrosarcoma and mesothelioma; the latter exhibiting metastasis throughout the abdomen. Tumors were consistent with the types of sarcomas commonly seen in individuals affected with LFS – mainly bone, CNS and soft tissue (Malkin et al., 1990).

### F344-*Tp53* osteosarcomas were common with frequent pulmonary metastases

**Fig. 4.**
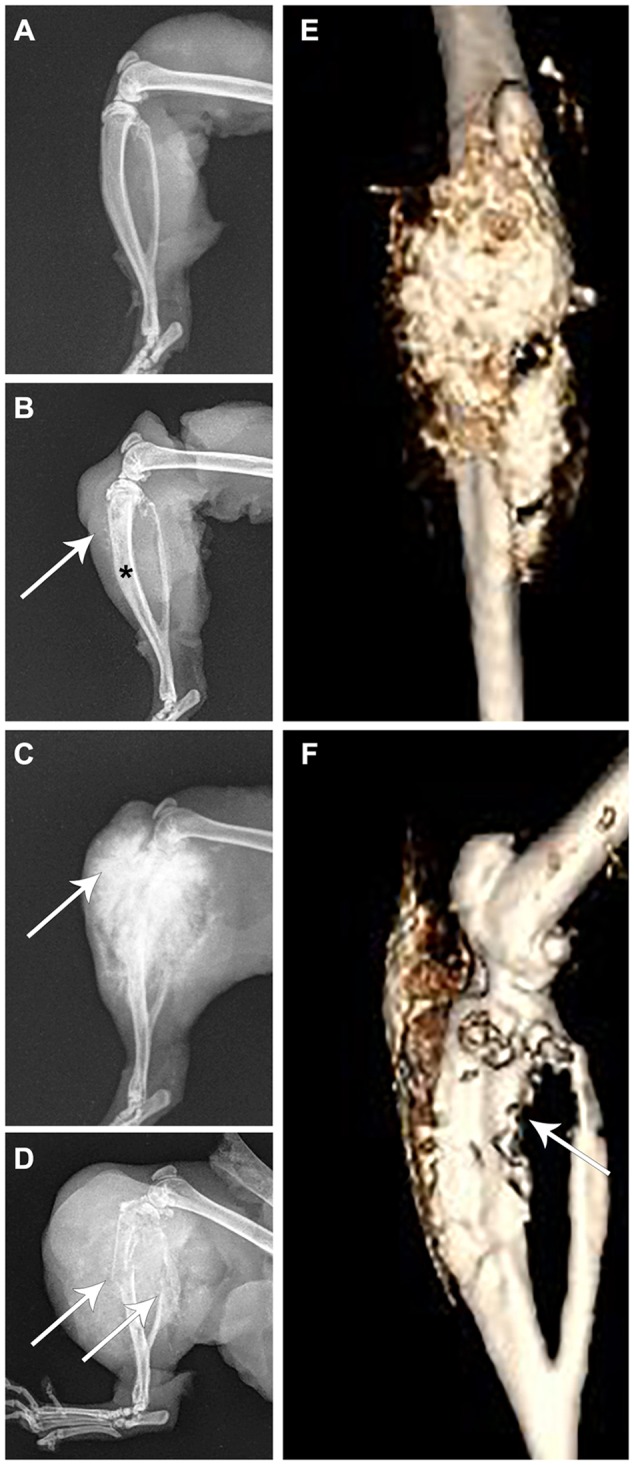
**Imaging of osteosarcomas in the F344-*Tp53* rat.** Rat limbs from four animals. (A) A normal healthy limb. (B,E,F) Different images of the same limb of an animal euthanized for lethargy. (C,D) Limbs from two different rats euthanized because they showed hindlimb swelling and lameness with obvious tumor growth. (A) Unaffected rat tibia and fibula; (B) osteosarcoma of the tibia with loss of normal medullary cavity opacity (asterisk), minimal soft-tissue swelling and early osteoid deposition (arrow); (C) osteosarcoma of the tibia exhibiting a typical ‘sunburst’ pattern of abnormal osteoid deposition in the tumor (arrow); (D) osteosarcoma of the tibia exhibiting pathologic fracture of both the tibia and fibula (arrows), considerable soft-tissue swelling and osteoid deposition; (E) anterior-posterior view in an un-enhanced computed tomography image of the same osteosarcoma shown in B, revealing in more detail the extent of osteoid deposition (gold colored); and (F) lateral view in an un-enhanced computed tomography image of the same limb in B,E exhibiting cortical lysis of the tibia (arrow).

**Fig. 5.**
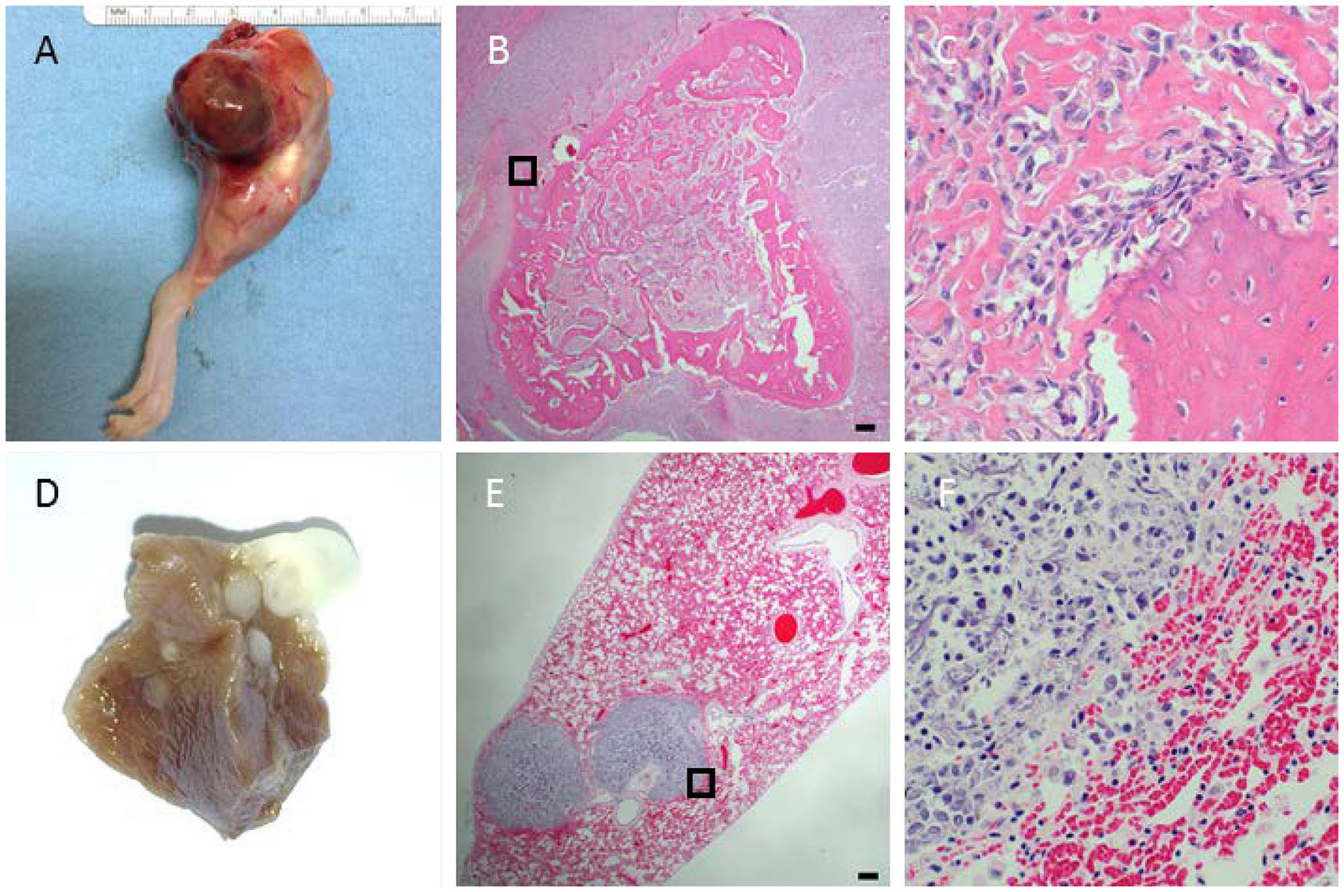
**Osteosarcoma histology.** Conventional, high-grade osteosarcomas were identified in F344-*Tp53* rats on long bones with frequent pulmonary metastases. (A) Bone tumor present on the distal femur with surrounding soft-tissue swelling. (B) Low magnification of a distal femur tumor with diffuse spiculated osteoid surrounded by hypercellular areas of spindle-shaped neoplastic cells. (C) Higher magnification of the boxed area in B demonstrating pleomorphic, osteoblastic spindloid neoplastic cells with production of osteoid matrix. (D) Multifocal ovoid raised mineralized lesions presenting as pulmonary metastases in fixed lung tissue. (E) Low-magnification image of perivascular metastatic neoplastic cell aggregates with foci of mineralization. (F) Higher magnification of the boxed area in E showing pleomorphic polygonal cells and spindle-shaped neoplastic cells with abundant basophilic strands of mineralization (osteoid). (B,C,E,F) Hematoxylin and eosin (H&E) stains. Scale bars: 150 µm; width of C and F represents 150 µm.

Osteosarcomas were identified in 57% and 36% of homozygous and heterozygous animals, respectively. F344 rats normally show a very low incidence of osteosarcomas, with 0.2-0.4% reported previously (Haseman et al., 1998). Most osteosarcomas were found on long bones, and resulted in rats presenting with lameness and limb swelling (Figs 4 and 5A). Tumors were identified on the proximal tibia, proximal humerus and distal femur. Radiographs of long-bone tumors exhibited considerable amounts of soft-tissue osteoid, starburst bone growth, normal bone destruction, ill-defined tumor margins and pathologic fractures (Fig. 4A-D). Bone tumors radiographically did not cross a joint and were limited to a single bone. Computed tomography analysis of hindlimb tumors revealed excessive soft-tissue osteoid development and bone destruction that was limited to the tibia (Fig. 4E,F). Exceptions in regards to long bone location included one tumor on the mandible and one on a lumbar vertebra; the rat with the latter presenting with ataxia that progressed to hindlimb paresis, which was worse on the right side. The tumor was identified on the right side of a lumbar vertebra. All osteosarcomas, with one exception, were high-grade and conventional in type, exhibiting the production of an immature osteoid matrix (Fig. 5B,C). The single exception was a limb tumor in a homozygous animal without osteoid matrix, consistent with a minimally osteogenic osteosarcoma or a fibroblastic subtype of osteosarcoma. Despite this tumor finding, osteosarcoma metastasis was present in the pulmonary parenchyma, indicating that the animal indeed harbored an osteosarcoma, although a slight variation from the other types. Each homozygous and 4 out of 9 heterozygous rats that presented with osteosarcoma also exhibited histopathologic evidence of pulmonary metastasis. Metastases could be identified grossly in some animals as large firm nodules of osteoid present within and on the surface of the pulmonary parenchyma (Fig. 5D). In other animals, pulmonary metastasis was identified histologically, with osteoid present throughout large perivascular regions of neoplastic cells (Fig. 5E,F).

### Meningeal sarcomas are a predominant tumor in animals presenting with lethargy or sudden death

**Fig. 6.**
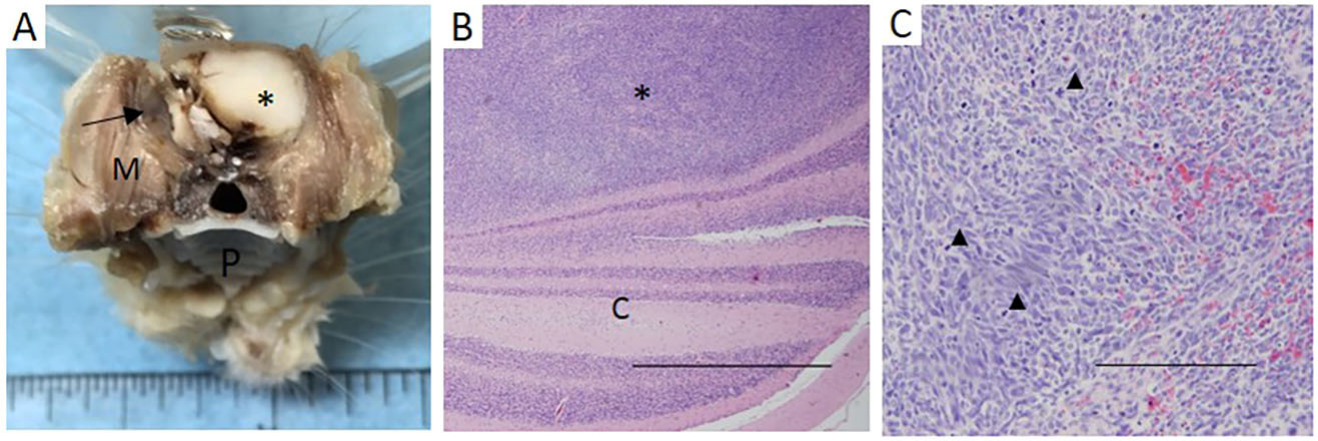
**Meningeal sarcoma histology.** Representative meningeal sarcoma. (A) Expansile meningeal sarcoma (asterisk) *in situ* affected the cerebral cortex (fixed tissue, normal brain indicated with arrow; M, masseter muscle; P, hard palate). (B) Meningeal sarcoma (asterisk) invading into the cerebellum (‘C’). Scale bar: 1 mm. (C) Higher magnification of meningeal sarcoma with characteristic spindloid neoplastic cells with high mitotic index (arrow heads indicate mitotic figures). Scale bar: 250 µm.

Brain tumors, specifically meningeal sarcomas, were identified in 6 out of 7 homozygous animals. The brain of the seventh rat was not available for evaluation owing to post-mortem autolysis following sudden unexpected death. Meningeal sarcomas were also identified in heterozygous animals, but to a lesser extent (7 out of 25 rats). Tumors were highly invasive, with extensive disruption of normal brain tissue (Fig. 6A-C). Animals with meningeal sarcomas were frequently found to either die suddenly after appearing clinically normal the previous day or they would present with depressed or dull mentation.

### Other clinical observations

Three animals were euthanized based on clinical signs of illness or distress in the absence of observable tumors. The single female homozygous rat required euthanasia owing to dystocia. Following histopathologic evaluation, this female was identified to have a meningeal sarcoma. Interestingly, *Tp53* homozygous mice have been noted previously to have an increased rate of dystocia (Donehower, 1996; Hirota et al., 2010). Two heterozygous rats developed lethargy and dull mentation with one being diagnosed post-mortem with pyelitis and pyelonephritis, and the other having no identifiable tumors upon histopathologic evaluation.

## DISCUSSION

### Advantages of rat models for biomedical research

For years, aging and carcinogenicity studies have been performed in the well-characterized F344 rat, with data on over 100,000 study animals archived with the National Cancer Institute and National Toxicology Program (ntp.niehs.nih.gov). These studies provide a vast amount of historical data on the Fischer strain, including rates of spontaneously occurring neoplasms and average life span under various housing and disease conditions. In addition, the rat's increased body size is amenable to studies requiring invasive techniques and repeated sampling, such as device testing, serum biomarker detection and metabolomics. With the recent advances in the ability to manipulate the rat genome, it is now possible to generate a wide variety of genetically engineered rats (Bao et al., 2015; Geurts et al., 2009; Mashimo, 2014). The size, physiology and genetics of the rat, along with the ability to manipulate the genome, position the rat to be the premier species for modeling human disease.

### Importance of genetic background

The use of outbred stocks in drug and toxicology studies is often justified as a means to mimic human genetic diversity. However, commonly used breeding strategies for genetically modified animals created on an outbred background rarely maintain this genetic diversity. In addition, closely linked loci surrounding the genetic modification can become fixed and have an unappreciated effect on phenotype. By generating or maintaining mutations on an inbred genetic background, phenotypic variability due to genetic variation can be avoided.

### F344-*Tp53* provides a refined model for the study of cancer

Similar to previous studies showing phenotypic variability in mice with *Tp53*-null mutations on various genetic backgrounds, transfer of the null *Tp53* mutation from the original outbred STOCK to the inbred F344 rat results in a distinct phenotype shift (Aitman et al., 2008; Donehower et al., 1995). Although both strains primarily developed sarcomas, the F344-*Tp53* rat had tendency to form bone and brain tumors, compared to the more frequent liver and blood neoplasia identified in the STOCK-*Tp53* rat. This is likely to represent a direct effect of the genetic background on the tumor spectrum. Further genetic studies could identify candidate modifier genes that might explain the genotype-to-phenotype variation. A major advantage of the F344-*Tp53* congenic is the ability to model osteosarcoma and other tumor types on a standardized inbred genetic background.

### Sex bias in homozygous mutants

Although the *Tp53*-null state is not considered embryonically lethal, the finding of fewer *Tp53* homozygous females is not surprising based on literature reporting lower recovery rates of *Tp53*-null female mice due to the occurrence of exencephaly (Armstrong et al., 1995; Sah et al., 1995). Likewise, it has been noted that fewer homozygous females were recovered in two previously published *Tp53* rat models (McCoy et al., 2013; van Boxtel et al., 2011). It was shown in *Tp53*-null rats that the lack of live-born female homozygotes is due, at least in part, to neural tube defects that lead to embryonic lethality (Kawamata and Ochiya, 2012). This effect of *Tp53* indicates its importance not only in prevention of tumors but in its role in basic embryonic development.

### F344-*Tp53* osteosarcomas recapitulate human disease

The F344-*Tp53* rat is beneficial as it allows the study of bone tumors that develop in a tissue microenvironment more similar to that of humans, as the rat exhibits true lamellar bone structure, a feature lacking in the mouse. Owing to the genetic complexity of osteosarcoma development, it is difficult to model tumor initiation and progression (Hansen et al., 1985; Sampson et al., 2013; Poos et al., 2015). Previous studies have used significant manipulations – such as long-term parathyroid hormone [PTH(1-34)] administration or tumor transplantation in the soft-tissue environments – or have utilized immunocompromised animals (Schwartz et al., 2013; Tsukamoto et al., 2012; Vahle et al., 2002). The tumors that developed in our rats recapitulate the aggressive human osteosarcoma phenotype, with tumors that primarily affect long bones and that readily metastasize. This is in direct contrast to mouse osteosarcomas that develop primarily on the axial skeleton and metastasize about 20% of the time (Walkley et al., 2008). Additionally, as we have shown, imaging of tumor-bearing rats can be performed to monitor for tumor occurrence and growth, as well as metastasis development, and to evaluate treatment efficacy in drug trials. The original STOCK-*Tp53* rat has been reported to have several osteosarcomas based on histological appearance; it has not been reported whether pulmonary metastasis of these tumors occurs. In our study, 57% of F344-*Tp53*^−/−^ and 36% of *Tp53*^+/−^ rats had osteosarcomas, with pulmonary metastasis occurring in 100% and 44% of these animals, respectively. Although previously reported in 18% of Sprague-Dawley *Tp53^Δ11/Δ11^* rats and up to 67% in the Crl:WI(UL)-Tp53m1/Hubr heterozygous rats, the F344-*Tp53* rat showed elevated rates of osteosarcoma in both the homozygous and heterozygous states, making it a promising model for deeper investigation into osteosarcomagenesis (Hermsen et al., 2015; McCoy et al., 2013; van Boxtel et al., 2011).

### Meningeal sarcomas are a prevalent tumor type in the F344-*Tp53* model

The presence of meningeal sarcomas in 86% of homozygous and 24% of heterozygous animals is of interest in that the F344 inbred strain itself has a low – 0.1% – spontaneous occurrence rate of this type of tumor (Haseman et al., 1998). Of the previous *Tp53*-null rat models, the Crl:WI(UL)-Tp53m1/Hubr and STOCK-*Tp53* rat had no reported brain tumors. In contrast, the Sprague-Dawley *Tp53^Δ11/Δ11^* rat had a brain tumor incidence of 42%, many of which were reported as meningeal sarcomas with a very similar histologic appearance to what was seen in our F344-*Tp53* rats. In humans, meningeal sarcomas are rare and aggressive tumors that typically affect children (Buttner et al., 2001). The meningeal sarcomas in our model were histologically similar to human tumors in that they feature small round uniform cells with scant cytoplasm (Jing et al., 2014).

In summary, the F344-*Tp53* knockout rat is highly suitable for oncology research. The mutation is now present in an inbred strain of rat frequently utilized in research. The inbred genetic background allows for easy maintenance of the model and introgression of additional mutations that already exist on the F344 background. The inbred F344-*Tp53* knockout rat is a powerful model to investigate compelling questions surrounding the development of osteosarcomas and meningeal sarcomas.

## MATERIALS AND METHODS

### Ethical statement

All activities associated with this study received prior protocol approval by the University of Missouri Institutional Animal Care and Use Committee. The University of Missouri is United States Department of Agriculture (USDA) licensed and Association for Assessment and Accreditation of Laboratory Animal Care (AAALAC) accredited.

### Housing and husbandry

Rats were housed in micro-isolator cages on ventilated racks (Thoren, Hazleton, PA) in an environmentally controlled animal facility maintained at 68-79°F, 30-70% humidity on a 14:10-h light:dark cycle in accordance with recommendations set forth in the Guide for the Care and Use of Laboratory Animals, 8th Edition (Animal Welfare Act, Animal Welfare Regulations, ILAR Guide). Breeders were fed PicoLab 5058 breeder chow with *ad libitum* access to acidified autoclaved water. Study animals were fed PicoLab 5008 chow. Routine health monitoring was performed by Charles River (Wilmington, MA).

### Animals

The strain characterized in this study is F344-*Tp53^tm1(EGFP-Pac)Qly^*/Rrrc, available from the Rat Resource and Research Center (RRRC), University of Missouri (RRRC# 661). This strain carries a *Tp53* allele in which exons 2-5 have been replaced with a CAG-EGFP-IRES-Pac cassette (Tong et al., 2010). A marker-assisted speed congenic approach was used to generate the strain. Male rats from STOCK-*Tp53^tm1(EGFP-Pac)Qly^*/Rrrc generated by Qi-Long Ying (USC) (Tong et al., 2010) and available through the RRRC (RRRC# 485) were backcrossed to F344/NHsd females from Harlan Laboratories (Indianapolis, IN). The resulting offspring were genotyped for the *Tp53^tm1(EGFP-Pac)^* allele, and heterozygous males were backcrossed to female F344/NHsd rats. For each successive generation, microsatellite analysis using a low-density panel of markers was performed, as previously described, on DNA from heterozygous males to identify those rats with the highest percentage of tested loci that were homozygous for F344 alleles (Bryda and Riley, 2008). These males were used to produce the next generation. Heterozygous animals were intercrossed to generate homozygous animals. All rats used in the study were from generations N5-7.

### Genotyping for the *Tp53* allele

DNA from tail-snip biopsies that had been collected from 3-week-old rats was extracted using the QIAGEN DNeasy Blood and Tissue Kit (QIAGEN, Valencia, CA) with minor modifications to the manufacturer's protocol as follows: AW1 and AW2 wash steps were repeated once and followed by one elution into 200 μl of AE buffer. The primers used to identify the wild-type and *Tp53^tm1(EGFP-Pac)^* alleles were 5′-GCGTTGCTCTGATGGTGAC-3′, 5′-GGGAGGATTGGGAAGACAATAGC-3′ and 5′-CAGCGTGATGATGGTAAGGAT-3′. PCR was performed in 20-μl reactions containing: 10-20 ng genomic DNA, 2 μl 10× buffer with MgCl_2_ (Roche, Indianapolis, IN), 3.2 μl of 1.25 mM dNTP mix (Promega, Madison, WI), 0.3 μl each of 25 μM primers and 1 U FastStart Taq DNA polymerase (Roche). Reactions were performed in 200-μl thin-walled PCR tubes, and the thermocycling parameters were: 1 cycle at 95°C for 5 min, 35 cycles of 94°C for 30 s, 63°C for 30 s, 72°C for 30 s, and 1 cycle at 72°C for 10 min. Amplicons of 310 bp (wild-type allele) and 509 bp (mutant allele) were produced. Products were analyzed using the QIAxcel Advanced capillary electrophoresis system with the QIAxcel DNA Screening Kit, QX Alignment Marker 15 bp/1 kb, XQ DNA Size Marker 50 bp-800 bp. The AM320 Injection Method with injection for 10 s at 5 kV and separation of 320× at 6 kV was used.

### Endpoints

Rats were monitored daily for morbidity, as defined by: palpable tumors, lethargy, dyspnea, abnormal mentation or a combination of clinical signs of disease. When morbidity was noted, rats were euthanized.

### Histopathology and phenotyping

Rats were euthanized by CO_2_ inhalation and subjected to a routine necropsy for gross evaluation of tissues and tumor collection. The following tissues were harvested and evaluated from each animal: any suspected masses, tumors or lesions, brain, heart, lung, liver, kidney, adrenal gland, pancreas and spleen, and any limb affected with lameness or swelling. Lesions were recorded, and tumor samples were dissected and submitted for routine histopathology preparation. Briefly, soft tissues were immersed in 10% buffered formalin for 48 h and processed for histological examination. Tumors that were attached to bone were fixed in 10% buffered formalin for 48 h followed by decalcification with a 50:50 solution of sodium citrate and formic acid until samples could easily be sectioned with a No. 20 scalpel blade. All tissues were processed for paraffin embedding, blocked in wax and cut into 5-μm sections, which were stained with hematoxylin and eosin. Samples were evaluated by using brightfield microscopy in a blinded study method to determine tumor classification.

### Imaging

Radiographs were obtained of long-bone tumors following tissue fixation but before decalcification. Tabletop images were obtained on an Innovet classic X-ray unit (Innovet, Chicago, IL) utilizing 42 kVP and 0.83 mAs. Computed tomography was performed following isoflurane anesthesia on a Toshiba 64-slice Aquilion scanner with Quantum De-noising Software (QDS) (Toshiba, Tustin, CA).

### Statistical methods

Chi-squared statistical analyses were performed using the Mstat 6.1.1 software package (http://mcardle.wisc.edu/mstat/).
